# A novel untargeted metabolomics correlation-based network analysis incorporating human metabolic reconstructions

**DOI:** 10.1186/1752-0509-7-107

**Published:** 2013-10-23

**Authors:** Helen L Kotze, Emily G Armitage, Kieran J Sharkey, James W Allwood, Warwick B Dunn, Kaye J Williams, Royston Goodacre

**Affiliations:** 1 School of Chemical Engineering and Analytical Science, Manchester Institute of Biotechnology, University of Manchester, Manchester M1 7DN, UK; 2 Current address: Centre for Metabolomics and Bioanalysis (CEMBIO), Facultad de Farmacia, Campus Monteprincipe, Universidad CEU San Pablo, Boadilla del Monte, Madrid 28668, Spain; 3 Department of Mathematical Sciences, The University of Liverpool, Peach Street, Liverpool L69 7ZL, UK; 4 School of Chemistry, Manchester Institute of Biotechnology, University of Manchester, Manchester M1 7DN, UK; 5 Current address: School of Biosciences, University of Birmingham, Edgbaston, Birmingham B15 2TT, UK; 6 Centre for Endocrinology and Diabetes, Institute of Human Development, The University of Manchester, Manchester M13 9PL, UK; 7 Centre for Advanced Discovery and Experimental Therapeutics (CADET), Central Manchester University Hospitals NHS Foundation Trust, York Place, off Oxford Road, Manchester M13 9WL, UK; 8 Manchester Pharmacy School, University of Manchester, Oxford Road, Manchester M13 9PT, UK

**Keywords:** Metabolomics, Correlation analysis, Network analysis, Cancer, Hypoxia

## Abstract

**Background:**

Metabolomics has become increasingly popular in the study of disease phenotypes and molecular pathophysiology. One branch of metabolomics that encompasses the high-throughput screening of cellular metabolism is metabolic profiling. In the present study, the metabolic profiles of different tumour cells from colorectal carcinoma and breast adenocarcinoma were exposed to hypoxic and normoxic conditions and these have been compared to reveal the potential metabolic effects of hypoxia on the biochemistry of the tumour cells; this may contribute to their survival in oxygen compromised environments. In an attempt to analyse the complex interactions between metabolites beyond routine univariate and multivariate data analysis methods, correlation analysis has been integrated with a human metabolic reconstruction to reveal connections between pathways that are associated with normoxic or hypoxic oxygen environments.

**Results:**

Correlation analysis has revealed statistically significant connections between metabolites, where differences in correlations between cells exposed to different oxygen levels have been highlighted as markers of hypoxic metabolism in cancer. Network mapping onto reconstructed human metabolic models is a novel addition to correlation analysis. Correlated metabolites have been mapped onto the Edinburgh human metabolic network (EHMN) with the aim of interlinking metabolites found to be regulated in a similar fashion in response to oxygen. This revealed novel pathways within the metabolic network that may be key to tumour cell survival at low oxygen. Results show that the metabolic responses to lowering oxygen availability can be conserved or specific to a particular cell line. Network-based correlation analysis identified conserved metabolites including malate, pyruvate, 2-oxoglutarate, glutamate and fructose-6-phosphate. In this way, this method has revealed metabolites not previously linked, or less well recognised, with respect to hypoxia before. Lactate fermentation is one of the key themes discussed in the field of hypoxia; however, malate, pyruvate, 2-oxoglutarate, glutamate and fructose-6-phosphate, which are connected by a single pathway, may provide a more significant marker of hypoxia in cancer.

**Conclusions:**

Metabolic networks generated for each cell line were compared to identify conserved metabolite pathway responses to low oxygen environments. Furthermore, we believe this methodology will have general application within metabolomics.

## Background

Within systems biology, metabolomic studies have been shown to be an essential component for elucidating the interdependency between substrates and products. To date, metabolomics data have been limited to relatively standard statistical techniques including univariate analyses and multivariate analyses such as principal component analysis [1,2]. Whilst these have shown potential to elucidate disease or cellular phenotypes and to discover potential predictive biomarkers, their ability to provide insights into the underlying biochemical function is limited, particularly when a system is in a perturbed state through disease or environmental stress. In this scenario, a deeper insight into a system response could be offered using novel computational approaches to allow improved interpretability of the complex large-scale metabolomic datasets generated.

The concentrations of metabolites in cells relate in part to the underlying structure of the metabolic network and thus the relative concentrations of metabolites within these networks can exhibit correlations [3]. There have been examples of correlation analysis for metabolomics that have allowed powerful biological interpretation of data [4]. Although a simple interpretation of metabolite correlations would be to assume that strongly correlated metabolites must be neighbours in the metabolic network, in reality this is often not the case. For example, metabolites that are spatial or temporal neighbours may not be strongly correlated whilst apparently distant metabolites are correlated [3,5]. Valcarcel *et al*. recently demonstrated the application of differential correlation analysis to create networks offering an insight into characterising a variety of biological states [6]. Whilst these networks provided a snapshot of the system response and were useful to determine differences between individuals with different phenotypes, in this case normal fasting glucose and pre-hyperinsulinameia, the underlying origin of the correlation with respect to metabolic pathways was not explored. Bridging the gap between analysing large-scale untargeted metabolomics data and interpreting the biological regulation in relation to the entire metabolic network may benefit from combining genome-scale metabolic networks. There are several large-scale human metabolic networks available, however popular models include the global reconstruction of the human metabolic network based on genomic and bibliomic data reconstruction 1 (recon1) [7] and the recently published recon2 [8] as well as the Edinburgh human metabolic network (EHMN) [9]. The EHMN is available as both compartmentalised or un-compartmentalised [10]. Depending on the samples and the application, compartmentalised or un-compartmentalised forms offer different advantages. For example, if the biological samples used for the analysis are organelle specific, more specific information may be gained from using a compartmentalised model; whereas if the samples are representative of whole cells and the overall cellular phenotype is of interest then using an un-compartmentalised model can be more accurate. These models have been used previously, for example recon1 was used to predict alternative drug targets for treating hyperlipidaemia [7].

In this study a network-based correlation analysis method has been developed as a novel method for the identification of linked metabolic pathways in the context of the entire metabolic network. Pair-wise correlations were mapped onto the EHMN genome-scale metabolic network and the shortest path connecting the two metabolites was extracted and used to construct a network of the cellular response to a physiological perturbation. Since all reactions present in the EHMN are reversible, all correlations were considered reversible and in this way it does not matter in which direction the path was drawn between correlated metabolites. Network biology is an emerging field; however there are complex issues, which remain to be addressed. For example, the shortest path between metabolites has been previously described for *Escherichia coli*[11]. The average path connecting metabolites was shown to be longer than paths found by treating the metabolic system as a normal network. Furthermore, there is the added influence that nodes may affect one another along multiple paths simultaneously [12].

We shall illustrate this method for investigating tumour hypoxia. Cancer metabolism has fascinated biologists since Warburg’s experiments in the 1920s [13]. Otto Warburg was the first to note that tumour cells rely on anaerobic metabolism as a source of energy production even under aerobic physiological oxygen levels [14]. Hypoxia is prevalent in solid tumours and is a feature associated with the aggressive disease and poor response to therapy. It arises and persists in tumorous tissue due to poor oxygen supply and rapid oxygen consumption. Hypoxia generally occurs in cells that are located at a distance greater than 100 – 180 μm from the blood supply [15]. Rapid cell proliferation and the presence of abnormal blood vessels in tumours contribute to creating distances greater than this threshold, thus causing hypoxia [15]. Hypoxia can be simulated in cancer cells such that its effect on cell metabolism can be studied at the simplest level. Several metabolomic studies have revealed metabolic features of hypoxia in cancer using *in vitro* cell models [16,17]. Here a similar approach has been applied to study the role of metabolism in hypoxia, using a non-targeted metabolite profiling approach to gain a global profile of the metabolome of each sample (*via*. cell-environment interaction). Rather than simply to analyse the data in terms of each single metabolic feature that changes with respect to oxygen level, we have applied our novel correlation mapping onto the EHMN to reveal both correlated metabolites whose concentrations change in unison and anti-correlated discordant metabolites.

## Results and discussion

### Correlation analysis

Two tumour cell lines cultured *in vitro* and exposed to 21% oxygen (normoxia) or 1% oxygen (hypoxia) have been compared to assess the metabolic effect of lowering oxygen availability and to determine whether or not these effects are commonly observed across different cell lines. The cell lines used were the human breast carcinoma cell line MDA-MB-231 and the human colon carcinoma cell line HCT116. Cell lysates were prepared for each cell line exposed to each experimental oxygen condition and metabolites were profiled using gas chromatography–mass spectrometry (GC-MS). For each cell line exposed to each condition, a total of 30 replicates were collected. Over 50 metabolites were detected using GC-MS in each cell line exposed to normoxia or hypoxia (see Table 1). Some of the metabolites detected were not uniquely identified as a single metabolite, typically as a consequence of isomerisation. Multiple identifications are often the result of the high-throughput nature of the metabolic profiling method where some metabolites with a similar chemical structure cannot be chromatographically resolved.

**Table 1:** 52 metabolite peaks detected using gas chromatography mass spectrometry (GC-MS) including corresponding KEGG ID or associated pathway

**ID**	**Metabolite name**	**KEGG ID**	**Pathway**
1	Glycine	C00037	Amino acid metabolism
2	Lactate	C00186	Glycolysis pathway
3	Pyruvate	C00022	Glycolysis pathway
4	Valine	C00183	Amino acid metabolism
5	Leucine	C00123	Amino acid metabolism
6	Glycerol	C00116	Glycerolipid Metabolism
7	Isoleucine	C00407	Amino acid metabolism
8	Leucine	C00123	Amino acid metabolism
9	Malonate	C00383	Pyrimidine metabolism
10	Glycine	C00037	Amino acid metabolism
11	Phosphate	C00009	Osmolyte, enzyme cofactor, signalling
12	Threonine	C00188	Amino acid metabolism
13	Alanine	C00041	Amino acid metabolism
14	Threonine	C00188	Amino acid metabolism
15	Succinate	C00042	TCA cycle
16	Benzoic acid	C00180	Unknown
17	Threitol/erythritol	C00503	Unknown
18	Malate	C00149	TCA cycle
19	4-hydroxyproline	C01157	Amino acid metabolism
20	Aspartate	C00049	Amino acid metabolism
21	4-aminobutyric acid	C00334	Amino acid metabolism
22	Aspartate	C00049	Amino acid metabolism
23	4-hydroxyproline	C01157	Amino acid metabolism
24	Xylitol	C00379	Pentose and glucuronate interconversion metabolism
25	2-hydroxyglutaric acid	C03196	Butanoate metabolism
26	4-hydroxybenzoic acid	C00156	Carbohydrate metabolism
27	Methionine	C00073	Amino acid metabolism
28	Creatinine	C00791	Amino acid metabolism
29	Putrescine	C00134	Amino acid metabolism
30	Hypotaurine	C00519	Amino acid metabolism
31	Glutamate	C00025	Amino acid metabolism
32	2-oxoglutarate	C00026	TCA cycle
33	Fructose	C02336	Carbohydrate metabolism
34	Sorbose/fructose	-	Carbohydrate metabolism
35	Sorbitol/galactose /glucose	-	Carbohydrate metabolism
36	Sorbose/fructose	-	Carbohydrate metabolism
37	Glycerol 3-phosphate	C00093	Glycolysis pathway
38	Galactose/glucose	-	Carbohydrate metabolism
39	Galactose/glucose	-	Carbohydrate metabolism
40	Galactose/glucose	-	Carbohydrate metabolism
41	Citrate	C00158	TCA cycle
42	N-acetyl aspartate	C01042	Amino acid metabolism
43	Glucose	C00031	Carbohydrate metabolism
44	Scyllo-inositol	C06153	Carbohydrate metabolism
45	Lysine	C00047	Amino acid metabolism
46	Myo-inositol	-	Carbohydrate metabolism
47	Pantothenic acid	C00864	Pantothenate and CoA biosynthesis
48	Tyramine/tyrosine	-	Amino acid metabolism
49	Hexadecanoic acid	C00249	Fatty acid metabolism
50	Octadecanoic acid	C01530	Fatty acid metabolism
51	Myo-inositol phosphate	C01177	Carbohydrate metabolism
52	Lactose/maltose	-	Carbohydrate metabolism

Those metabolites that possess a KEGG identifier are those that have been definitively identified to Level of MSI [18].

**Figure 1:**
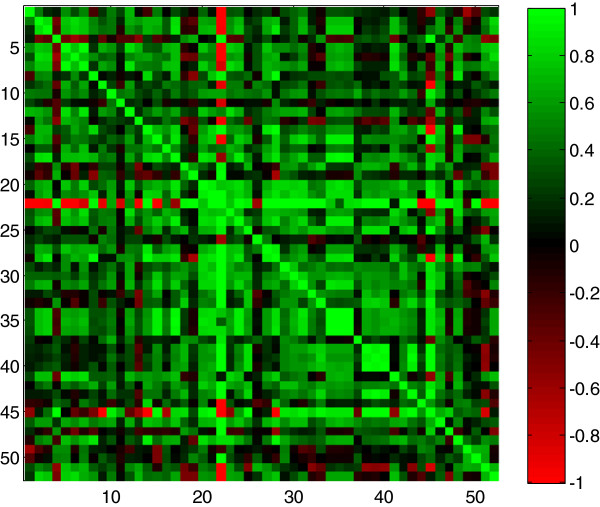
**A colour heatmap of the Pearson’s correlation coefficients computed for the 52 metabolites observed in the MDA-MB-231 cells exposed to normoxia (21% oxygen).** The metabolites appear in the same order as in Table 1. The colours refer to the pair-wise correlation coefficient ranging from 1 (green) to -1 (red).

Using these identified metabolites, the approach was to reveal correlations between the metabolites that differed between the two oxygen levels. Statistical outliers were identified within each experimental group as values greater than three standard deviations away from the mean for that metabolite in that group [19]. These were replaced with a mean and the data were observed to follow an approximately normal distribution, meeting the requirements for the Pearson’s correlation method. For each experimental group of 30 replicates in turn, a Pearson’s correlation coefficient was computed between metabolites in a pair-wise fashion. An example of the calculated correlation coefficients for metabolites detected in the MDA-MB-231 cell line cultured in normoxia are shown as a heatmap in Figure 1, where green represents a positive correlation and red a negative correlation. Different methods for correlation analysis can be employed and the decision is usually based upon the type and quality of the data. For example, the Pearson’s product–moment correlation method computes a coefficient for normally distributed data and the relationship is linear [5], or at least assumed to be. Since our data were observed to follow an approximately normal distribution, it was appropriate to perform the Pearson’s product–moment correlation method. When such requirements are not met it may be more appropriate to use a routine that does not use mean values in correlations such as the Spearman’s rank correlation analysis.

In the MDA-MB-231 model, a total of 7 correlations were found to differ significantly in response to lowering oxygen availability. For the HCT116 cell line a total of 18 correlations were significantly different between normoxia and hypoxia. The five most significantly different correlations between normoxia and hypoxia are shown in Table 2. for MDA-MB-231 samples and in Table 3 for HCT116 samples. For HCT116 all correlations are positive in hypoxia which suggests the response to low oxygen is to up-regulate rescue pathways. By contrast the MDA-MB-231 cells appear to no longer correlate glucose/malate metabolites and rather correlate malate/pyruvate and octadecanoic acid/glutamate to promote cell survival.

**Table 2:** Metabolite pairs that were differentially correlated between normoxia and hypoxia in MDA-MB-231 samples

**Metabolite A**	**Metabolite B**	**Correlation coefficient (normoxia)**	**Correlation coefficient (hypoxia)**	**Correlation coefficient difference**
Glucose	Malate	0.701	-0.420	1.125
Galactose/Glucose	Malate	0.708	-0.262	0.975
Malate	Pyruvate	-0.139	0.725	0.865
Octadecanoic acid	Glutamate	-0.068	0.717	0.791
Glucose	Galactose/ Glucose	0.922	0.291	0.617

**Table 3:** Metabolite pairs that were differentially correlated between normoxia and hypoxia in HCT116 samples

**Metabolite A**	**Metabolite B**	**Correlation coefficient (normoxia)**	**Correlation coefficient (hypoxia)**	**Correlation coefficient difference**
Galactose/Glucose	4-Hydroxyproline	0.082	0.886	0.804
4-Hydroxyproline	Malate	0.199	0.902	0.702
4-Hydroxyproline	Aspartate	0.287	0.951	0.664
Fructose	4-Hydroxyproline	0.124	0.753	0.629
4-Hydroxyproline	Glycerol	0.200	0.794	0.594

The oxygen response for MDA-MB-231 cells cultured in normoxia shows a correlation between glucose and malate, glucose/galactose and malate and glucose and glucose/galactose. Furthermore, for MDA-MB-231 cells cultured in hypoxia there is a correlation between malate and pyruvate and octadecanoic acid and glutamate. The shift from malate being correlated with glucose in normoxia to then being correlated with pyruvate in hypoxia may represent a shift from oxidative phosphorylation metabolism to partial use of the tricarboxylic acid (TCA) cycle though glutaminolysis. The TCA cycle is part of central carbon metabolism and it is widely known to be one of the main responses to hypoxia [20]. Glutaminolysis has been suggested to be an important energy source in tumour cells [21]. Hypoxic cells may utilise this pathway when low oxygen availability causes a shift from oxidative phosphorylation metabolism to non-oxidative phosphorylation metabolism. Consequently, glutaminolysis partially uses the TCA cycle resulting in the production of malate and pyruvate. This pathway can contribute towards cellular survival in low oxygenated environments through the generation of ATP, NADH and synthesis of metabolic precursors such as amino acids and nucleotides required for cell growth. Furthermore, acetyl-CoA entering the TCA cycle can be consequently guided towards *de novo* synthesis of fatty acids for cell growth. Glutamate and octadecanoic acid are immunosuppressive metabolites that are often released by the cells to protect the system from an immunity attack [22,23]. The correlation between these two metabolites may be a consequence of the glutaminolysis pathway increasing the production of these two metabolites. Potentially interfering with the pathways that connect these pair-wise correlated metabolites could lead to selectively killing the hypoxic tumour and furthermore, network mapping may help to identify effective novel targets for drugs.

Although it has not previously been linked with hypoxia, the greatest difference in correlation between HCT116 cells exposed to normoxia and hypoxia occurred in the correlation between galactose/glucose and 4-hydroxyproline. These metabolites are correlated in hypoxia but are not correlated in normoxia and must therefore be a response to hypoxia. Furthermore, the 4-hydroxyproline peak was correlated with many other metabolites in hypoxia and could be potentially considered a central 'hub’ in cellular response to this oxygen condition, such that pathways involving this metabolite are changed in regulation. This highlights the potential importance of this metabolite in a hypoxic response.

Many of the metabolites found to be differently correlated between normoxia and hypoxia in both cell lines were features of central carbon metabolism. Such metabolites include glucose, malate and pyruvate. Central carbon metabolism is obviously a feature of hypoxic response that is conserved between cell lines; however, the way the concentrations of these metabolites is affected by hypoxia is to a certain extent cell line specific. Other central carbon metabolites may also have changed in the same way within the system, just that they were not detected. It is possible to measure all central metabolites and they are present in our libraries, however many can be missing from the profile if their concentrations are below the limits of detection [24].

### Network analysis

The metabolic profiles acquired for correlation analysis here were obtained from whole cell lysates of MDA-MB-231 and HCT116 human carcinoma cells. For this reason it was appropriate to use a metabolic reconstruction that did not contain compartmentalisation of metabolites, for example those biochemical reactions that occur in the cytosol and those that are contained in the mitochondria. The EHMN contains approximately 2800 non-compartmentalised reactions [9]. Correlated metabolites were identified within the EHMN and the shortest path between these was computed from the reactions available in the network. This allowed the connection between correlated metabolites to be observed. From a list of differently correlated metabolites between two experimental groups at a time it was possible to collect pathways between them and together create new sub-networks to describe the network based origin of the differences. Some metabolite pathways could not be extracted from the EHMN as the metabolite was not present in the network or the metabolites were not connected in the network. This is a result of the limitation of the reconstructed human metabolic networks as some reactions of human metabolism are currently unknown. The future constructions of more detailed metabolic networks may overcome this; note that Recon2 [8] may address this but was not available during this study. Subsequently, pathways from each correlation were combined to generate a network to visualise the significant difference. The aim was to use the networks to observe variation and similarities between the cell lines with respect to lowering oxygen availability. The EHMN and other large-scale networks contain highly connected nodes from molecules such as ATP, H_2_O and NAD. When computing the shortest network path connecting two nodes the path calculates a shortcut that passes through these highly connected nodes [25]. These molecules are present in many of the biochemical reactions as products or substrates; however they are side reactions (co-factors) and therefore these intermediates were removed prior to applying graph-theory.

**Figure 2:**
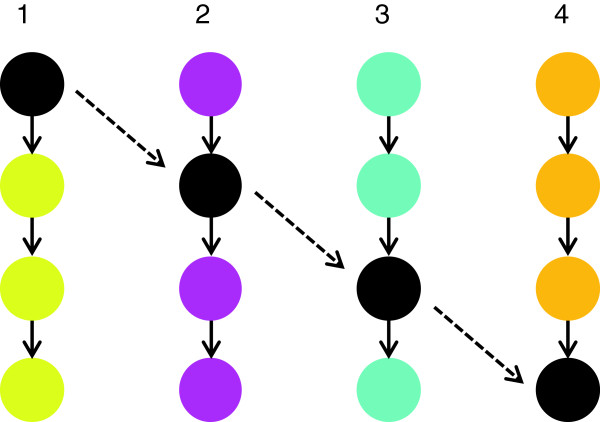
**An alternative way to view metabolic pathways.** Metabolism involves many inter-connections between metabolites; however there are traditional ways to represent pathways**.** In this schematic **1**, **2**, **3** and **4** represent 4 individual pathways as they are traditionally considered, however a pathway exists in metabolism that can connect these 4 pathways *via* the intermediates of each. This pathway (highlighted in black) could biochemically be more important than **1**, **2**, **3** or **4**.

Metabolism is usually considered in terms of pathways in the way they are traditionally represented in databases such as KEGG. Many of the pathways in these databases are interconnected and it is possible to consider new pathways that cross over several 'traditional’ pathways that may potentially be more biochemically relevant than each of the traditional pathways; this is depicted as a cartoon in Figure 2. The method used to build sub-networks of differently correlated metabolites in this research has found interlinked pathways that connect correlated metabolites *via* the shortest route. This means many pathways form interactions between traditional pathways and thus cross over them (the black route in Figure 2). Sub-networks have been built considering metabolic pathways in this different way and although this is less conventional, it has made it possible to reveal potentially more relevant pathways than if only 'traditional’ pathways were considered. This is demonstrated in Figure 2 which is comprised of real biochemical reactions but that are not illustrated in this way in traditional pathways such as in KEGG.

**Figure 3:**
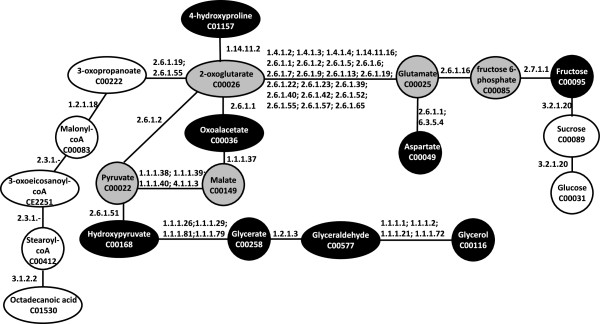
**Network of pathways connecting differently correlated metabolites between normoxia and hypoxia in both MDA-MB-231and HCT116 cell lines.** Nodes unique to MDA-MB-231 cells are shown in white, nodes unique to HCT116 cells are shown in black, and nodes common between cell lines are shown in grey. The KEGG identification code is given for each metabolite listed along with the enzymes used for each reaction.

Figure 3 shows a network of pathways connecting differently correlated metabolites in normoxia and hypoxia for cell lines MDA-MB-231 (white) and HCT116 (black); where metabolites common in both the cell lines are shown in grey. This global view of both cell lines enables the comparative study of the metabolic connectivity. The grey nodes could potentially be central metabolites, marking a change in metabolism in response to hypoxia that is qualitatively the same between the two cancer cell lines. The network encompasses a range of KEGG pathways including glycerolipid metabolism (ko00561); glycine, threonine and serine metabolism (ko00630); glycolysis/gluconeogenesis (ko00010); fructose and mannose metabolism (ko00051) and the TCA cycle (ko00020) to name a few. In this way, Figure 3 describes how the underlying mechanisms of a real biological system can be more complex than considering individual pathways in isolation as they are traditionally represented.

From this network in Figure 3 it can be seen that malate, pyruvate, 2-oxoglutarate, glutamate and fructose-6-phosphate are conserved features of metabolic response to hypoxia in both MDA-MB-231 and HCT116. These five metabolites can be connected *via* a single pathway between malate and fructose-6-phosphate using pyruvate, 2-oxoglutarate and glutamate as intermediates. However the network also shows that all five of these metabolites interact with other metabolites *via* different reactions. They are central to this network depicted in Figure 3 and describe differential metabolic regulation between normoxia and hypoxia. The shortest network path connecting these metabolites occurs in the TCA cycle. As previously suggested for MDA-MB-231 cells, the hypoxia cells may be producing glutamate as an immunosuppressive response which would protect the hypoxic cells from immune attack. This metabolite is shared in the network and it may be that the HCT116 cells also synthesise glutamate as an immunosuppressive response through an alternative mechanism to glutaminolysis [23]. Furthermore, glutamate is also a precursor for nucleic acid synthesis and may have the potential to drive the uptake of other amino acids from the extracellular environment [26].

Hypoxic cells are expected to rely on increasing non-oxidative phosphorylation metabolism to promote survival due to the decreasing oxygen availability limiting oxidative phosphorylation metabolism. One of the interesting observations of the network (Figure 3) is that lactate does not feature in the network suggesting non-oxidative metabolism is not an important feature of cellular survival at 1% oxygen compared to cells cultured in normal oxygen levels. It may be that this process is induced at lower than 1% oxygen and therefore was not observed in this experiment. The network suggests that cells are using mechanisms other than non-oxidative phosphorylation metabolism to promote survival such as shunting off into the TCA cycle.

## Conclusions

Within systems biology, metabolomics is an essential tool as it allows the establishment of interdependency between metabolites. Metabolic profiling generates large-scale datasets, with complexity that makes interpretation difficult with respect to providing an insight into cell physiology. Here, the network visualisation of the biochemical connectivity between metabolite concentrations using correlation analysis has been demonstrated as a novel method to generate hypotheses about the regulation and cellular response to an environmental perturbation in the context of systems biology. The topological differences in the network offer a further dimension to the understanding of the regulation of key metabolites.

Network-based correlation analysis offers a complementary tool to statistical and multivariate analysis methods typically applied to metabolomics datasets to identify metabolite differences in physiological state. Additionally, this method could be readily applied to metabolic profiling data from different biological systems or to other profiling datasets such as those acquired in proteomics and transcriptomics. Its application in systems biology could therefore be of paramount importance.

## Methods

### Experimental

#### Cell lines and cell culture

Human cell lines HCT116 colorectal carcinoma and MDA-MB-231 breast adenocarcinoma were routinely cultured in RPMI 1640 (Gibco BRL, Paisley, UK) supplemented with 10% fetal calf serum (Labtech International, East Sussex, UK) from a single batch and 2 mM glutamine (Sigma-Aldrich, Dorset, UK). Cells were incubated in 95% air and 5% CO_2_ at 37°C and 95% relative humidity.

#### Treatment

8 mL of cells were seeded, in exponential phase, at 1 × 10^6^ cells/mL into a 10 cm^2^ Petri dish (Falcon, Runcorn, UK). For consistency, the same batch of undefined FCS was used for all experiments. Cells were cultured and allowed to adhere in normoxia (21% oxygen) for 24 h prior to experimental analysis in the incubator described above. Subsequently, cells were divided into two groups where one group was retained in the normoxia condition and the other group was placed in a closed vessel though which gas containing 1% O_2_, 5% CO_2_ balanced with N_2_ was flowed at a rate 2 mL/s (hypoxia). This vessel was developed in-house and was constructed from an ADDIS® 5 L air tight container (including removable clip lid) with a male BSPT- female BSPP reducer and male parallel straight adaptor to connect a 6 mm polytube to a 6 mm 2/2 finger tap at either side of the container (all components were sourced from RS Components Ltd, Stockport, UK).

#### Metabolite extraction protocol

Prior to metabolite extraction, the hypoxic vessel was closed using the taps and placed in an anoxic chamber (Bactron anaerobic chamber, Sheldon Manufacturing, Cornelius, Oregon, USA). Normoxic samples were extracted in air. Extracellular media were decanted and cells were washed three times with 1 mL phosphate buffered saline (PBS). Subsequently, 1 mL methanol (maintained at -48°C) was added to quench cellular metabolism. Cells were scraped from the culture surface and the suspension solution was placed into Eppendorf tubes. A series of three freeze thaw cycles using liquid nitrogen were performed to enhance metabolite extraction before which the solution was centrifuged (17000 × *g* for 15 min) and the supernatant was transferred into a fresh Eppendorf tube. The volume of supernatant to be lyophilised was normalised according to the weight of the pellet for intracellular samples. Furthermore, supernatant remaining after normalisation was used to generate QCs which contained an equal proportion from each sample. Supernatant for both the sample and QC were lyophilised.

#### Metabolite profiling by GC-MS

Prior to analysis, all samples were chemically derivatised. In the first stage of derivatisation, 50 μL of a 20 mg/mL solution of O-methoxylamine in pyridine was added to each sample, ensuring the pellet of metabolites was fully immersed. Each sample was then vortexed in this solution and heated at 60°C for 30 min. The second stage of the process involved adding 50 μL of N-methyl-N-(trimethylsilyl trifluoroacetamide (MSTFA) to each sample which was then vortexed and heated at 60°C for 30 min. Finally, samples were centrifuged at 17000 × *g* for 10 min to pellet the debris and 20 μL of a retention index marker solution was added which contained 0.3 mg/mL n-decane, n-dodecane and n-pentadecane, n-docosane and n-nonadecane in pyridine. The resulting supernatant from each sample was collected for analysis.

Samples were analysed using an Agilent 6890 GC (Agilent Technologies, Stockport, UK) coupled to a LECO Pegasus III (Leco Corp., St. Joseph, MO) EI-ToF-MS. The GC-MS instrument setup used has been previously described [27,28]. The acquisition run was formed to start with a derivatisation blank then 5 QC samples (as previously optimised [28]) followed by 5 samples followed by another QC followed by another 5 samples and so on until the end of the analysis. The temperature was set at 70°C for 4 min followed by a 20°C increase every min until 300°C was reached and stabilised for 4 min. Samples were injected (2 μL) onto the column. The total duration to analyse a single sample was 25 min.

The processing of raw GC-MS data was performed following the methods described previously, using the LECO ChromaToF v3.25 software package to apply the chromatographic deconvolution algorithm [27]. Identifying the metabolites within the compiled database was performed through searching against an in-house mass spectral and retention index library as described previously [24], where a mass spectral match greater than 80% and a retention index match ± 20 provided a definitive identification. Metabolites that were not identified in the in-house database were searched against the Golm metabolome database [29]. The level of identification reported was applied according to reporting guidelines as described by the Metabolomics Standards Initiative [30].

#### Data analysis

Data were normalised to the internal standard succinic acid *d*_*4*_*.* QC metabolites with a CV greater than 30% were removed from the whole data matrix [28]. Prior to statistical analysis outliers were replaced with the mean of the group which were identified as metabolites with a ratio of more than three deviations away from the mean group [19]. These data are given in Additional file 1. Each cell line is shown in a different sheet and sample types are labelled N, H and A corresponding to the normoxia, hypoxia and anoxia treatments.

The Pearson’s correlation calculation is given in Equation 1:

(1) r=∑i=1nXi-X¯Yi-Y¯∑Xi-X¯2∑Yi-Y¯212

Equation 1: The Pearson’s product–moment correlation equation (as shown in Rodgers and Nicewander (1988) [31]) where *r* is the correlation coefficient calculated for the pair-wise correlation of variables *X* and *Y*, $\bar{X}$ and $\bar{Y}$ are the mean values for variables *X* and *Y* respectively and *n* is the number of samples.

To test for a significant correlation, the correlation value was approximately normalised using the inverse transform of Equation 2 which is known as the Fishers $\dot{z}$ -transformation.

(2) $$\dot{z}=\frac{1}{2}\log\frac{1+C}{1+C}$$

Equation 2: Fishers $\dot{z}$ -transformation where *C* is the correlation coefficient (i.e., *r* from Equation 1), which approximately normalises the distribution of the correlation coefficient to a Gaussian distribution and is independent on the number of samples.

The significance of the correlation was tested using Equation 3.

(3) $${\hat{z}}^{T}={\dot{z}}^{T}\sqrt{N-3}$$

Equation 3: Determining the significance of the correlation that is dependent on sample number (*N*), where the distribution is approximately Gaussian and the 95% significance level is given by ${\hat{z}}^{T}$ ± 1.96 for α = 0.05 (see Table 4).

**Table 4:** **The value for**${\hat{z}}^{T}$**with a significance of *α*** [32]**used to calculate the correlation threshold *C***^***T ***^**that corresponds to the significance level *α***

***α***	**10**^**-6**^	**10**^**-5**^	**10**^**-4**^	**10**^**-3**^	**0.01**	**0.05**
${\hat{z}}^{T}$	4.891638	4.417117	3.890592	3.290527	2.575829	1.995996
*C*^*T*^ (*N* = 27)	0.760964	0.717096	0.660761337	0.586081	0.482156	0.380014

Results shown are calculated for an effective *C*^*T*^ for a sample size of 27.

Subsequently the correlation threshold was reported using the inverse transform of Equation 2 as shown in Equation 4 for a sample size of 27. Results are shown in Table 4 where the correlation threshold was calculated for a range of ${\hat{z}}^{T}$ values.

(4) $$C^{T}=\frac{e^{\left(2{\dot{z}}^{T}\right)-1}}{e^{\left(2{\dot{z}}^{T}\right)+1}}\mathrm{with}\;{\dot{z}}^{T}=\frac{{\hat{z}}^{T}}{\sqrt{N-3}}$$

Equation 4: The effective correlation coefficient generated from the inverse of Equation 2.

Since the correlation threshold was relatively low for *α* = 0.05 Equation 5 was applied to determine the minimum number of samples that would be required to accept correlations with a standard error less than or equal to 0.1. The minimum number of samples required to obtain a statistically significant correlation was determined to be 27. Consequently, a total of 30 biological replicates were extracted for each condition to minimise the biological variance in the dataset. In the event that correlation coefficients where computed where either metabolite contained less than 27 entries (due to missing values where either the metabolite was not present in the sample or it was present at a concentration not detectable by the GC-MS) these correlations were discarded.

(5) $$\mathrm{SE}=\frac{\left(1-\rho^{2}\right)}{\sqrt{n-1}}$$

Equation 5: The standard error equation where *SE* is the standard error, *ρ* is the correlation coefficient and *n* is the sample size.

For each metabolite detected, the GC peak area was used for correlation. The Pearson’s correlation coefficients between metabolites were computed pair-wise using Equation 1. Subsequently, the difference between the correlations for two sample types (e.g., the correlation difference for HCT116 samples exposed to normoxia or hypoxia) was calculated. To be considered a significant difference, one of the correlations must be > 0.7 and the difference between the correlations coefficients must be > 0.407. This was determined using the Fishers $\dot{z}$ -transformation and permutation test as shown in Equation 2 and Equation 6 respectively. The Fishers $\dot{z}$ -transformation approximately normalises the correlation coefficient to a Gaussian distribution. This was inputted into Equation 6 to calculate the difference required between two correlations for it to be considered significant. The significance at level *α* = 0.05 was tested using Equation 6 to satisfy the ${\hat{z}}^{T}$ = 1.96 in Table 4. In this case a correlation of 0.7 is accepted with a minimum of 27 samples and there must be a correlation difference of 0.407 to be statistically significant. This was performed using in-house routines in the software package Matlab version 9 software (The Mathworks, Inc., Natick, MA, USA).

(6) $$\left(a\right)\;{\dot{z}}_{i}=\frac{1}{2}\log\frac{1+C_{i}}{1+C_{i}}\;\left(b\right){\hat{z}}^{T}=\frac{\left|z_{1}-z_{2}\right|}{\sqrt{\frac{1}{N_{1}-3}+\frac{1}{N_{2}-3}}}$$

Equation 6: (a) Fishers $\dot{z}$ -transformation where *C*_*i*_ is the Pearson’s rank correlation coefficient for example, 0.7. (b) Permutation test for comparing correlations between metabolites, where ${\hat{z}}^{T}$ is a value that corresponds to the confidence of a correlation, *z*_1_ and *z*_2_ are the values calculated through equation 3a and *N*_1_ and *N*_2_ are the minimum sample sizes for each metabolite [33].

Pair-wise correlations that significantly differed with respect to sample treatment were mapped directly onto the uncompartmentalised EHMN. For this the pair-wise correlations were taken in turn and using graph theory the shortest pathway connecting these two metabolites was computed. This was done using in-house routines in Matlab. The network was imported into Matlab using the SBML toolbox [34]. Subsequently, the currency metabolites were removed. Currency metabolites were defined as highly connected metabolites of side reactions and included metabolites such as water, ATP, ADP and other co-factors. These compounds reduce the average path length connecting two metabolites and therefore the pathways extracted do not represent the connectivity of reactions. For example, water has 1083 metabolite connections and therefore needed to be omitted along with other highly connected energy and redox cofactors including ATP, ADP, AMP, NAD, NADH, NADP, NADPH, CoA, UTP, UDP, UMP, GTP, GDP, H_2_O, CO_2_, O_2_, orthophosphate and hydrogen [9,35].

A stoichiometric matrix of the metabolic network was constructed using the SBML toolbox. This matrix was used to extract the two separate matrices of metabolites consumed and produced. The consumed network was multiplied by the transpose of the produced to construct a metabolite connectivity network. The matrix was symmetrised to account for reversibility in the network. Graph theory was implemented to calculate the shortest pathway between two metabolites using the bioinformatics toolbox included in Matlab.

## Competing interests

The authors declare that they have no competing interests.

## Authors’ contributions

HK and EA carried out data acquisition, data analysis and wrote the manuscript. JA and WB assisted in the data acquisition. KS helped in the initial data analysis. RG and KW participated in the design, data interpretation and reviewing of manuscript. All authors read and approved the final manuscript.

## Supplementary Material

Additional file 1 GC-MS data after normalisation to internal standard and filtering based on QCs. Click here for file
